# Population Ecology and Genetic Diversity of the Invasive Alien Species *Procambarus clarkii* in Lake Trasimeno (Italy)

**DOI:** 10.3390/biology10101059

**Published:** 2021-10-18

**Authors:** Ambrosius Josef Martin Dörr, Melissa Scoparo, Irene Cardinali, Gianandrea La Porta, Barbara Caldaroni, Gabriele Magara, Matteo Pallottini, Roberta Selvaggi, Beniamino Cenci-Goga, Enzo Goretti, David Cappelletti, Hovirag Lancioni, Antonia Concetta Elia

**Affiliations:** 1 Department of Chemistry, Biology and Biotechnology, University of Perugia, Via Elce di Sotto 8, 06123 Perugia, Italy; ajmartindoerr@libero.it (A.J.M.D.); melissa.scoparo@libero.it (M.S.); cardinali_irene@libero.it (I.C.); barbara_caldaroni@libero.it (B.C.); gabriele.magara@studenti.unipg.it (G.M.); matteo.pallottini@unipg.it (M.P.); roberta.selvaggi@unipg.it (R.S.); enzo.goretti@unipg.it (E.G.); david.cappelletti@unipg.it (D.C.); hovirag.lancioni@unipg.it (H.L.); antonia.elia@unipg.it (A.C.E.); 2 Department of Veterinary Medicine, University of Perugia, Via San Costanzo 4, 06126 Perugia, Italy; beniamino.cencigoga@unipg.it

**Keywords:** shallow lakes, red swamp crayfish, genetic characterization, mitochondrial DNA variation, population structure

## Abstract

**Simple Summary:**

The population of *Procambarus clarkii* was surveyed twenty years after its introduction into Lake Trasimeno (Central Italy), investigating both ecological and genetic aspects. Our results confirmed that *P. clarkii* is well acclimatized with a stable population structure, mainly characterized by a unique mitochondrial lineage, suggesting that a single introduction event may have occurred in the lake, complemented by secondary events.

**Abstract:**

The deliberate or accidental introduction of invasive alien species (IAS) causes negative ecological and economic impacts altering ecosystem processes, imperiling native species and causing damage to human endeavors. A monthly monitoring program was performed in Lake Trasimeno (Central Italy) from July 2018 to July 2019 in order to provide an upgrade of the population ecology of *Procambarus clarkii* and to assess the genetic diversity by analyzing the relationships among mitochondrial DNA diversity. Our results confirmed that *P. clarkii* is well acclimatized in the lake, revealing a stable population structure favored by the resources and conditions typical of this ecosystem, which seem to be optimal for the maintenance of the species. Four distinct mitochondrial haplotypes were detected, but one of them was clearly overrepresented (76%), suggesting that a single predominant introduction event may have occurred in this area, likely followed by secondary events. The identification of the typical genetic variants provides a better understanding of the evolutionary scenarios of *P. clarkii* in this biotope and it can be helpful in management plans concerning the expanding populations of this invasive alien species.

## 1. Introduction

Invasive alien crayfish have moved outside their natural ranges leading to ecological threats and adverse economic impacts. The red swamp crayfish *Procambarus clarkii* (Girard, 1852) is widely spread across all continents, except for of Australia and Antarctica [1,2,3]. *P. clarkii* has a great ecological adaptability that allows it to colonize most fresh and brackish water environments [4,5]. Its biological plasticity and resistance to environmental pollution determines its extreme invasiveness, competitiveness and aggressiveness with native species, making it almost impossible to eradicate from a territory once established. Indeed, *P. clarkii* is able to survive in waters with low oxygen concentration and can even survive out of water for a period of more than two days with a very low relative humidity level (30% RH) and for more than a month at 100% RH [6]. In dry periods, the species digs deep burrows (40–90 cm) which are also useful during the most delicate phases of its life cycle (molting and reproduction). This species can live in a wide range of pH [7], salinity [8,9] and environmental contaminant conditions [10,11,12]. It is an omnivorous predator, opportunistic and generalist consumer and extremely active both day and night. It enters the trophic network [13,14] acting as a keystone species causing a significant decrease in biomass and richness of species located at lower trophic levels. In general, the red swamp crayfish exerts a predatory pressure at which the native species are often not adapted. Moreover, *P. clarkii* is an asymptomatic potential carrier of the crayfish plague (*Aphanomyces astaci*) and an importer of pathogenic microfungal taxa such as *Phoma glomerata* (Coelomycetes) potentially harmful to human health, flora and fauna [15,16]. *P. clarkii* is reported in the Delivering Alien Invasive Species Inventory for Europe (DAISIE) list as one of the 100 worst invasive alien species and it is on the list of species of European Union concern linked to the EU Regulation 1143/2014 on invasive alien species. Red swamp crayfish exerts undesirable impacts on natural water resources producing a loss of ecosystem services [17,18]. The first introduction of red swamp crayfish in Italy in northern Italy dates back to 1989 [19]. Before 1999, no populations of allochthonous freshwater crayfish were reported in Umbria (Italy). Since the beginning of 2000, the *P. clarkii* population has rapidly increased in Lake Trasimeno [20]. Introduction of invasive red crayfish species into this lake has caused an ecological imbalance by altering the structure of the existing communities.

Trasimeno is a shallow lake subjected to severe hydrological level fluctuations through the seasons [21,22]. It is well known that in shallow lakes, hydrological levels represent a pivotal abiotic factor for both ecosystem and biota. Changes in water-level regime may cause shifts between the turbid and the clear water and biota responds to these variations [23]. Previous studies showed that dynamics and population structure of *P. clarkii* can be modulated by changes in water temperature and Lake Trasimeno level [4,20]. In Trasimeno *P. clarkii* can reach 5 years of age [4,20], whereas the biological cycle in the autochthone habitat usually does not exceed 12–18 months [24]. Both sexes invest heavily in reproduction, using a large part of their energy [20,24]. This crustacean is one of the most prolific species of crayfish, which usually produces from 500–600 to 700–750 eggs per female [25,26] and two reproductive events a year can also occur: one in spring and one in autumn. Furthermore, females provide parental care to eggs and hatched offspring. Its high reproductive capacity and growth rate has favored the rapid expansion of *P. clarkii* in this shallow and mesotrophic lake, increasing the economy of the primary (professional fishermen) and tertiary Lake Trasimeno sector. Nowadays the species is well acclimatized in Lake Trasimeno and has also colonized the lakes of Piediluco, Corbara, Alviano and Pietrafitta and at least six other watercourses in the Umbria region.

It is well known that the human-mediated jump dispersal of *P. clarkii*, including the intentional or unintentional transport, combined with its natural expansion influenced its population structure and genetic diversity. To date, there are no investigations focusing on the genetic variation of this species in Lake Trasimeno. There are only few population genetic studies dealing with the red swap crayfish invasion in the European continent based on different molecular markers [27,28,29,30,31]. Mitochondrial DNA (mtDNA) is considered one of the most useful markers to evaluate population dynamics [32,33,34]. The uniparental inheritance, the high mutation rate and the large number of copies inside the cells, make the mtDNA a very easy and cheap tool to identify the population variations. Moreover, it can be helpful in developing management measures to control invasions and to prevent further introductions [35]. Understanding how introduced species widely spread in non-native areas represents the key to avoid threats and facilitate the development of techniques that should prevent the invasions of allochthonous species. The analysis of genetic variability, in both native and invaded areas, allows to reconstruct the main invasion dynamics and identify the origin areas, thus obtaining information about the number of introductions (single or multiple) and describing expansion patterns.

This study presents a comprehensive overview of the red swamp crayfish status in Lake Trasimeno, with the aim to revisit the population ecology of *P. clarkii* twenty years after its introduction into Lake Trasimeno. In particular, its main objectives were: (i) to assess whether changes in life-history traits of red swamp crayfish in the lake may have occurred through time; (ii) to describe the growth of *P. clarkii* as well as estimate the condition indexes of both sexes, such as abdominal index (TwB), hepatosomatic index (HIw) and gonadosomatic index (GSI); (iii) to investigate the influence of the lake water levels on the life cycle of the species; (iv) to assess the genetic diversity of *P. clarkii* populations in Lake Trasimeno, analyzing the relationships among their mitochondrial haplotypes. The present study is the first investigation of *P. clarkii* genetic variability in this basin, based on the most polymorphic mitochondrial marker (mtDNA control region). It points to reconstruct the invasion history of the red swamp crayfish in Lake Trasimeno and to understand how the natural and anthropogenic factors could have influenced this species. Indeed, identifying the typical genetic variants inhabiting this shallow lake can be helpful to test single versus multiple introduction events and potential bottleneck effects, in order to shed light on their origin and demographic trends. This genetic characterization based on the mitochondrial marker represents a new molecular approach, more informative for the detection of the genetic variability of *P. clarkii*, thus useful to detect and manage the species.

## 2. Materials and Methods

### 2.1. Study Site

Lake Trasimeno is located in the province of Perugia (Umbria, Central Italy) and is the largest Italian peninsular lake, with a surface of 126 km^2^ and an average depth of 4.5 m. This laminar and mesotrophic lake is characterized by a Mediterranean climate, with maximum rainfall in spring and minimum in autumn, causing significant hydrologic fluctuations [21]. It is a Regional Park of Umbria and in accordance with the Council Directive 92/43/EEC on the conservation of natural habitats and of wild fauna and flora the site is designed as a Special Area of Conservation (IT5210018) and a Special Protection Area (IT5210070).

### 2.2. Chemical-Physical Parameters of Lake Trasimeno

The hydrological level (±0.1 cm) was recorded daily (data provided by the Umbria Region, Soil Defense Service). Temperature, transparency, pH, dissolved oxygen, percentage of dissolved oxygen saturation and conductivity were detected monthly in parallel with sampling of *P. clarkii* by multiparametric probes for dissolved oxygen, pH, temperature and conductivity (550A DO and Model 63, YSI Environmental Incorporated, Yellow Springs, OH, USA) and Secchi disk (Ecology Labs, Perugia, Italy) for transparency.

### 2.3. Sampling of P. clarkii and Morphometric Data Collection

A total of 1820 specimens of *P. clarkii* (1200 males and 620 females) were sampled from July 2018 to July 2019 in a protected wildlife area in the south-east part of Lake Trasimeno. A mean number of 92 males and 47 females were monthly collected from the lake by a professional fisherman within 24 h using fyke nets. Monthly samples were immediately transported to the laboratory and chill-killed using an ice-water bath. Morphometric parameters were recorded for both sexes in order to evaluate growth in length and weight and the crayfish condition indexes. Each crayfish was sexed and the following morphometric and biological parameters were measured:

- total body length (from the tip of the rostrum to the posterior margin of the abdomen (TL)
- length of the cephalothorax or carapace (CL)
- total body weight (W)
- abdomen weight (AW)
- hepatopancreas weight (HIw)
- mature gonad weight (for females only)

Length of total body and cephalothorax (with *rostrum*) were measured by a digital caliper to the nearest 1 mm; wet weights of total body, carapace, abdomen, hepatopancreas and gonad were measured using an electronic balance within 0.001 g accuracy. The sexual activity in males was assessed by observing the presence or absence of the spines on the third and fourth walking legs. Sexual maturity of females was assessed by observing the internal egg stages [20]. Ovarian egg color was used as an indicator of successive growth in oocytes: white color indicates the ovary is at rest (immature), yellow when the eggs have started maturation, orange when are almost mature, and dark brown when are mature and can be expulsed [4,20]. Crayfish carapace was analyzed by touch and specimens with a soft exoskeleton were identified as molted as well as those presenting slightly consumed gastroliths in their stomach [4].

### 2.4. Sex Ratio and Condition Indexes

*Sex ratio* and condition indexes such as abdominal (TwB) and hepatosomatic index (HIw) in both sexes, as well as and gonadosomatic index in females (GSI) were estimated using the following formulas [4,20]. Hepatosomatic Index (HIw) was carried out only for the whole male sample and females with mature gonads.

Sex ratio:

F:M = X:1

*F = females; M = males*

Hepatosomatic Index:

HIw = WH wet × 100/Wt

*WH wet = wet weight (g) of hepatopancreas*

*Wt = weight (g) of the body*

Abdomen Index Tw/B (Tail Muscle to wet body weight ratio):

Tw/B = WT wet ×100/Wt

*WT wet = wet weight (g) of abdomen*

*Wt = weight (g) of the body*

Gonadosomatic index:

GSI = [Wg/(Wt − Wg)] × 100

*Wg = wet weight (g) of the gonads*

*Wt = weight (g) of the body*

### 2.5. Statistical Analysis

Data were processed using the statistical programming environment R [36] and dplyr packages [37], tidyr [38] and ggplot2 [39]. In order to verify differences between the biometric variables of males and females, the Student t-test was performed, using a statistical significance level of 0.05. When necessary, data were Box-Cox transformed after testing for normality using the Shapiro-Wilk test. Comparisons with counting data were made using the chi-squared hypothesis test and correlations estimated by Persons’ product-moment coefficient.

### 2.6. Genetic Analysis

A total of 29 specimens were analyzed in order to determine DNA content and to sequence the mitochondrial control region. Genomic DNA was extracted both from the crayfish tail muscle and hepatopancreas using the Wizard^®^ Genomic DNA Purification Kit (Promega Corporation, Madison, WI, USA). A good quality of DNA was obtained from both kind of tissue with different concentrations expressed in ng/μL. The primers used for PCR amplification were those published by Li et al. [40] and the reaction conditions were as follows: initial denaturation at 95 °C for 2 min, followed by 35 cycles of denaturation at 95 °C for 30 s, annealing at 55 °C for 1 min and elongation at 72 °C for 1 min, followed by final extension at 72 °C for 10 min. After enzymatic purification (ExoSAP, USB), the PCR products were sent to Eurofins Genomics (Ebersberg, Germany) for Sanger sequencing using the following primer specifically designed: 5′-CTTCTAAAAATGTTCCCCCC-3′. The resulting sequences showed a variable length; they were aligned through the Sequencher software (www.genecodes.com accessed on 12 February 2021) together with those retrieved from GenBank [40] and compared to a sequence considered as reference mitogenome (NC_016926.1; [41]). In order to make uniform all sequences, they were cut at the same range, from the nucleotide position(np) 4717 to np 5434.

Mitochondrial DNA sequence variation parameters were estimated by using DnaSP 5.1 software (www.ub.edu/dnasp/index_v5.html accessed on 13 June 2021) [42]. Haplotype (HT) number codes have been assigned to each sample only for our convenience, as previously classified.

The evolutionary relationships among haplotypes were visualized through Molecular Evolutionary Genetics Analysis Integrated software (MEGA7; http://www.megasoftware.net accessed on 17 June 2021) by using the Neighbor-Joining algorithm, also including the mtDNA sequences available from GenBank.

## 3. Results

### 3.1. Chemical-Physical Parameters of Lake Trasimeno

**Figure 1:**
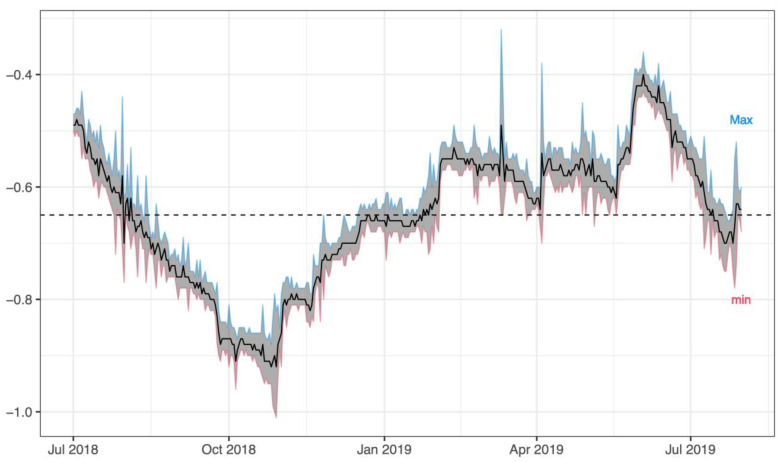
Hydrometric level of Lake Trasimeno during the monitoring period (July 2018–July 2019).

Hydrological levels of Lake Trasimeno were higher during spring and summer, with a maximum monthly mean of −0.47 m in June 2019. The lower values were recorded in October 2018 (−0.89 m) (Figure 1).

Seasonal chemical and physical parameters of Lake Trasimeno, such as water temperature, transparency, pH, dissolved oxygen, oxygen saturation and conductivity, are reported in Table 1.

### 3.2. Morphometric and Biological Analysis

#### 3.2.1. Sex Ratio and Population Size

**Figure 2:**
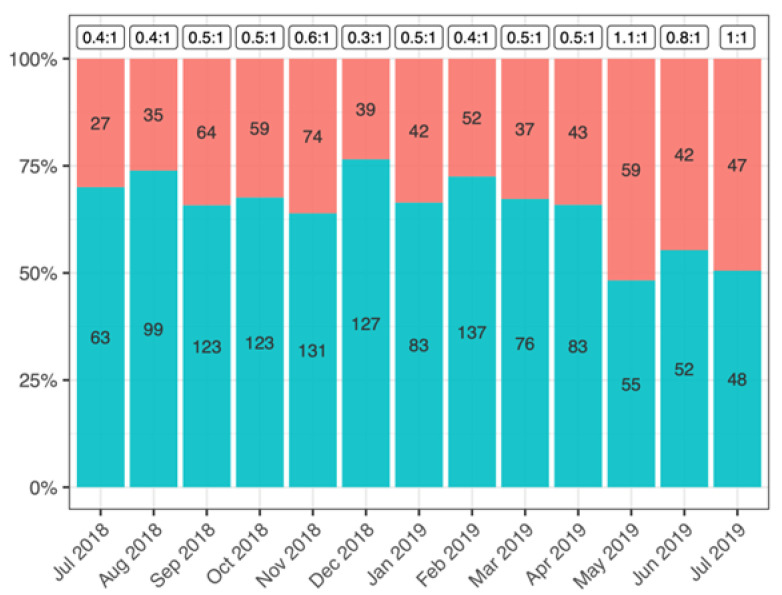
Sex-ratio and frequency of female (pink) and male (blue) crayfish from Lake Trasimeno (absolute values of both sexes inside the bars).

Generally, the number of males was statistically significant higher than that of females (Χ^2^ = 22.64; *p* < 0.05), accounting for 60–80% for most every monthly sample, excepted May, June and July 2019 showing a *sex ratio* close to 1:1 (Χ^2^ = 0.40; *p* > 0.05) (Figure 2).

Size (TL) and weight (W) of males were generally smaller than for females (Table 2). Mean total length of females was 99.46 mm (mean weight 23.06 g), with a minimum value of 45.03 mm and a maximum of 164.8 mm, while for males the mean value was 94.02 mm (mean weight 22.44 g, with a minimum of 65.91 mm and a maximum of 138.5 mm (t_TL_ = 8.7, df = 1792, *p* < 0.001; t_W_ = 0.49, df = 1817, *p* = 0.2) (Table 2).

The total mean of carapace length (CL) in females was 48.59 mm (min 20.14 mm; max 73.81 mm), and in males 46.76 mm (min 21.2 mm; max 81.55 mm). Higher carapace length was recorded during the warm months in females, with a maximum mean of 54.33 mm in July 2019, and lower in the colder months, with a minimum mean of 44.59 mm in November 2018. In males the carapace length slightly increased during the monitoring period, from a minimum mean value of 43.31 mm in September 2018 to a maximum value of 53.02 mm in June 2019 (tCL = 5.6, df = 1818, *p* < 0.001). For both sexes a statistically significant linear relationship between total length and total weight emerged (rfemales = 0.96, df = 601, *p* < 0.01; rmales = 0.89, df = 1188, *p* < 0.01).

#### 3.2.2. Molting and Reproductive Cycle

**Figure 3:**
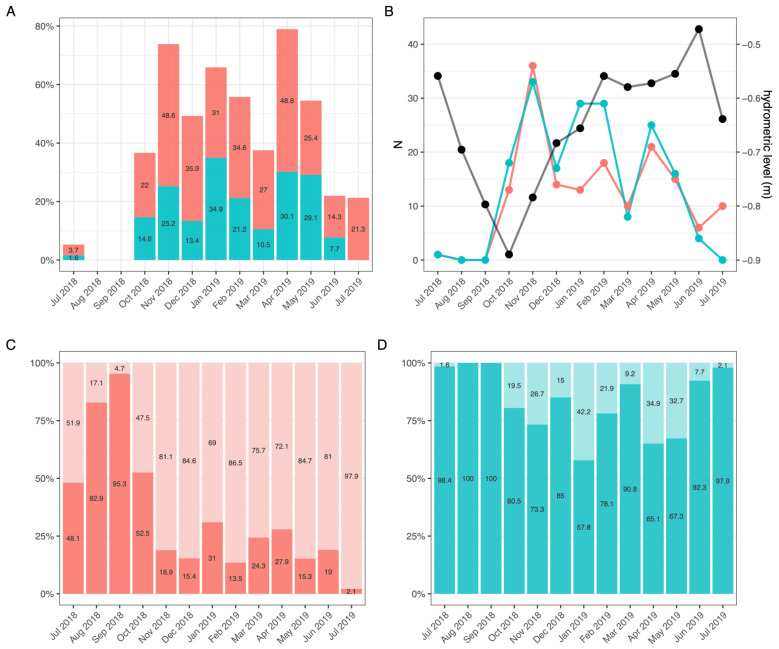
Frequency (**A**) and number of molts in relation to the hydrometric levels of Lake Trasimeno (**B**) in females (pink) and males (blue). Frequency of sexual maturity (percent values inside the bars) of females with mature (red) and immature (pink) gonads (**C**) and of sexually active (blue) and immature (light blue) males (**D**) of *P. clarkii* from Lake Trasimeno.

Molting (Figure 3A,B) occurred from late-autumn to late-spring, with a greater peak in November 2018 corresponding to the lowest value of hydrological level and the other two peaks in January-February 2019 and April 2019, especially in males, when lake level raised (Figure 3B). However, no statistically-significant correlation was found between lake water level and molting (r_females_ = −0.12, df = 11, *P* = 0.7; r_males_ = −0.14, df = 11, *p* = 0.65). Frequency of molts (Figure 3A) was higher in females, and seasonal fluctuations were inversely proportional to the sexual maturity observed in both sexes (r_females_ = −0.69, df = 11, *p* < 0.001; r_males_ = −0.99, df = 11, *p* < 0.001). In females, the maximum percentage of molting was recorded in November 2018 (48.6%) and April 2019 (48.8%). In males, a higher molting frequency (34.9%) was observed in January 2019, corresponding to the lowest percentage of sexual activity (57.8%, Figure 3A). July displayed the lowest frequency and no molts were registered in August and September. The smallest female with mature ovarian eggs had a total length of 70.24 mm (7.0 g), while the largest measured 139.58 mm (63.7 g). In August and September 2018 almost all adult females (82.9% and 95.2%, respectively) had mature ovarian eggs (Figure 3C), when the water temperature ranged between 18.7 and 25.7 °C, pH 8.7, dissolved oxygen 9.0 mg/L (110.5%), conductivity between 1607.33 and 1417.88 μS/cm and hydrometric level of about −0.75 m. No statistically-significant correlation was found between female abundance and maturity of ovarian eggs (r = 0.35, df = 11, *p* = 0.24). The frequency of sexually active males was generally high throughout the sampling months, except January, April and May 2019 (Figure 3D).

#### 3.2.3. Condition Indexes

**Figure 4:**
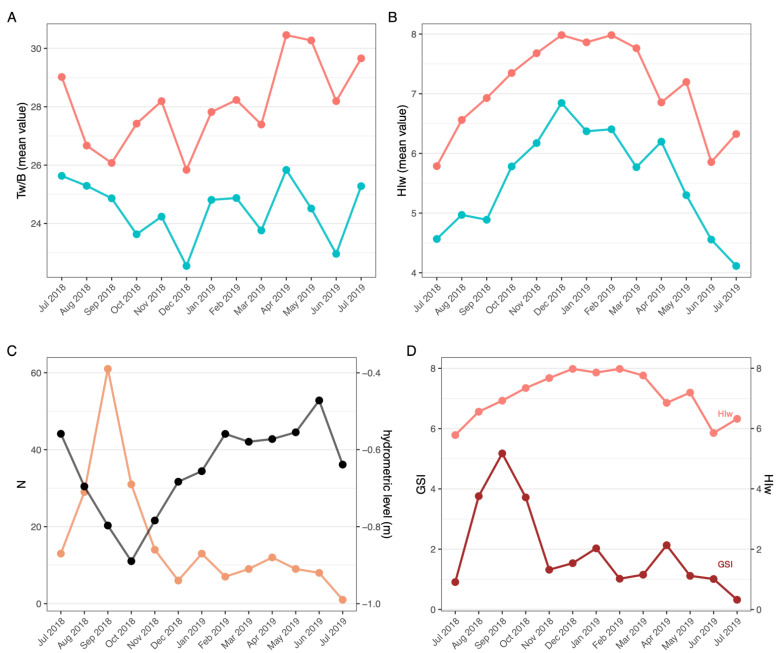
Tw/B index (**A**) and Hiw index (**B**) of females (pink) and males (blue) crayfish. Number of females with mature gonads (orange) in relation to the hydrometric levels of Lake Trasimeno (**C**) and HIw (pink) and GSI (brown) indexes of females (**D**) of *P. clarkii* from Lake Trasimeno.

Tw/B values in males were lower (5–20%) compared to those of females in each sampling month (Figure 4A). Similarly, a lower abdomen weight (up to 25%) was regularly measured in males (3–7 g and 3–8 g for males and females, respectively) (Table 2). In detail, the mean value recorded in females was 6.27 g (min 1.50 g; max 15.80 g), while in males 5.24 g (min 2.10 g; max 13.70 g) (t_AW_ = 8.8, df = 1818, *p* < 0.001). However, abdomen weight showed similar seasonal variations for both sexes, being higher from May to July and lower (up to 60%) in late summer and in colder months. A similar trend has also been observed for the Tw/B index. Lower HIw values were measured for males (10–35%) compared to females (Figure 4B). 

Indeed, for females the hepatopancreas mean weight was 1.61 g, with a minimum of 0.10 g and a maximum of 5.90 g, while for males the mean value was 1.25 g, with a minimum of 0.20 g and a maximum of 4.30 g (t_HW_ = 11, df = 1818, *p* < 0.001). Generally, hepatopancreas weight of most females was between 0.8 and 1.5 g, while for males it ranged between 0.8 and 1.2 g. Hepatopancreas weight, and consequently HIw, showed seasonal fluctuations, but in the opposite way to Tw/B (Figure 4A,B, Table 2). In females, the gonadosomatic index (GSI) differed during the sampling period, showing 3 peaks in September 2018 and January and April 2019 (Figure 4C). No correlation emerged between GSI and HIw (R = 0.042, df = 11, *p* = 0.89) (Figure 4D).

### 3.3. Genetic Analysis

A total of 718 bp of the mtDNA control region, spanning from nucleotide position (np) 4717 to np 5434, for each sample were analysed. The overall sequence alignment revealed the presence of 12 polymorphic sites (S), represented by any singleton variable sites of two variants, and 12 parsimony informative sites. The average number of nucleotide differences was 3.14. Nucleotide diversity (π) across all individuals was estimated at 0.00438, and the haplotype diversity (Hd) was determined at 0.414.

Both Tajima’s D nor Fu and Li’s D* values were negative and statistically significant: neither of neutrality test rejected the null hypothesis of neutral evolution (D = −0.07738, D* = −0.04512; *p* > 0.10); therefore, variation pattern of the mitochondrial control region may be a reliable reflection of the population history of *P. clarkii*. The negative Fs value (−0.01845) derived from sequence analyses was 0.773 (*p* = 0.393 > 0.05), thus suggesting that *P. clarkii* experienced a significant population expansion.

**Figure 5:**
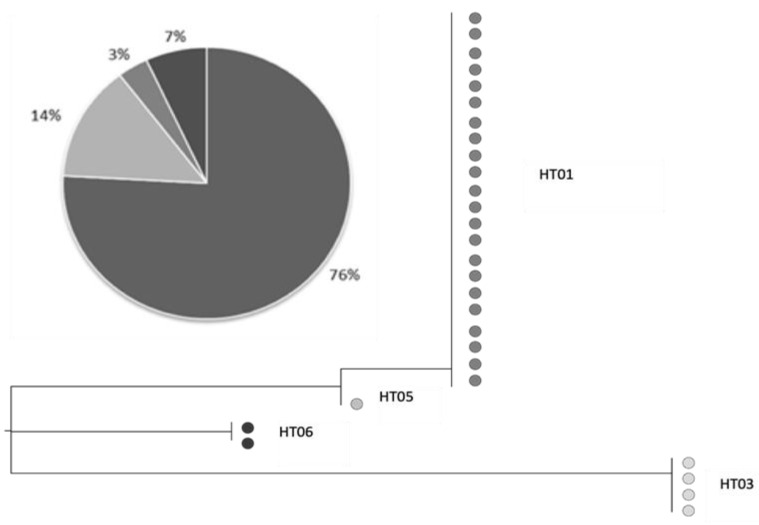
Pie chart of haplotype relative frequency and schematic representation of the mtDNA phylogeny of 29 samples from Lake Trasimeno.

Four distinct mtDNA haplotypes were detected (Table 3): HT01, HT03, HT05, and HT05. As reported in Figure 5, the most representative HT01 was detected in 22 out of 29 samples (76%), while one (HT05) was unique (3%). These two haplotypes were more closely related than others (Figure 5). HT03 was characterized by only four mutational variants relative to the reference sequence.

## 4. Discussion

It is well known that water column levels can influence the behavior and reproductive cycle of *P. clarkii* [4,20,43,44,45]. The levels in Lake Trasimeno have changed considerably over time; during the 1970–1975 and 1989–2012 periods the lake level decreased more than 1 m and 2 m below the hydrometric zero level, respectively, whereas during the rainy years 2013–2014 it increased considerably. Indeed, in order to prevent flooding of coastal areas, in January 2015 the bulkhead of the artificial outlet of San Savino was moved down after a total closure of about 30 years [21]. Although hydrological levels of Trasimeno increased considerably over the last five years, fluctuations of lake level during seasons lead to reach the highest level in spring and summer and the lowest level in autumn and mainly in October. These changes in the hydrological level require physiological adaptations of aquatic organisms [46]. Therefore, the present study assessed whether changes of life-history traits of the invasive red crayfish species occurred in the last decade in Lake Trasimeno.

The sex ratio throughout the monitoring period of the present study was generally different from the expected 1:1 previously found for cambarids [26] and for *P. clarkii* in Lake Trasimeno [20]. An imbalanced sex ratio in specimens from the same ecosystem has already been reported by Dörr and Scalici [4]. According to the authors, females have never prevailed over males in the years 2007–2009, differently from what was observed for *P. clarkii* eight years earlier [20].

In this study, conducted 10 years after the last study of Dörr and Scalici [4], a male/female ratio of 1:1 was recorded only in May, June and July 2019, while in the remaining period males were more abundant than females (60–80% of the entire monthly sample), in particular in August and December 2018 and February 2019. Moreover, the abundance of males has also been reported by other authors who have monitored the population of *P. clarkii* in Italian freshwater ecosystems [47,48,49].

Whereas in Dörr et al. [20] an inverse correlation between the abundance of females and the maturation of gonads emerged, the present study found no relationship between abundance and maturity of ovarian eggs of females. The sex ratio imbalance is likely due to the presence of several factors that can induce a different abundance between the two sexes, such as the breeding period, incubation period and digging habit [50,51,52]. In addition, other eco-ethological factors, such as the tendency of females, especially mature or with juveniles, to shelter in burrows [43,45], or the height of water column [44] could help to promote males abundance. It is quite difficult to attribute the imbalance of *sex ratio* to one or more biotic or abiotic factors. However, in this study, the increase in the hydrological level of the lake over the last five years may have played a pivotal role. The rise of the height of the water column may lead to an increase of the predation rate of *P. clarkii* in Trasimeno by its predators, such as largemouth bass (*Micropterus salmoides*), eel (*Anguilla anguilla*), pike (*Exos lucius*) and perch (*Perca fluviatilis*) [20]. At the same time the fishing pressure on *P. clarkii* by the professional fishermen may not be neglected in this basin.

The population of *P. clarkii* from Lake Trasimeno showed dimensions ranging from 6.2 cm to 14.0 cm of total length (5.90–65.0 g of total weight), mainly in a range between 8.5 to 10.5 cm. This outcome suggests that the red swamp crayfish is able to grow more in this shallow lake than in its original habitats [53,54,55]. However, the mean size of the *P. clarkii* population recorded in this lake seems to be slightly shortened since the beginning of the millennium [20].

The growth of crayfish is a process that involves a series of molting and inter-molting periods. Previous studies showed that molting period can be influenced by changes in hydrological levels and temperature of lake [4,20,26]. In the present study, molts were not strictly affected by the hydrological levels of Lake Trasimeno, even though the highest peak of crayfish in molt for both sexes was observed when the hydrological level was the lowest. Rather, molts showed seasonal variations and occurred mainly in autumn, winter and spring. However, although molting was predominantly in November 2018 and February and April 2019, the frequency was different in males and females, according to Dörr and Scalici [4]. Dörr et al. [20] found synchronized molting period for both sexes in spring. On the contrary, in the present study molting frequencies occurred asynchronously between sexes. While a single maximum molting peak was observed in males in January 2019, at a water temperature near to 5 °C, in females two peaks were recorded in different seasons: autumn (November 2018 at a water temperature near to 9 °C) and spring (April 2019, at a water temperature of about near to 17 °C).

For both sexes, molting activity significantly decreased in summer. Several published studies analyzed how temperature affects molting, some suggesting that it is favored at temperatures below 10 °C [56], others that the favorable period is when the temperature reaches 20 °C [54].

Molting is inversely related in both sexes to sexual maturity [20,57], which in turn is directly related to temperature, especially in females. In the crayfish monitored from July 2018 to July 2019, a correlation between molting and the reproductive stage of the specimens also emerged, with a higher molting frequency corresponding to a low percentage of sexually active males or females in the pre- or post-spawning phase (with immature ovarian eggs). On the other hand, reproductive cycle especially in females is related to temperature and hydrological levels. Previous studies have shown that the optimal growth temperatures of ovarian eggs range between 21 and 27 °C, while inhibition occurs below 12 °C [58]. In the present study, the peak of mature ovarian eggs occurred in September 2018, at a water temperature of 19 °C and a lower hydrological level (−0.8 m), after a progressive growth from July 2018, when the water reached higher temperatures (25–28 °C). These results are in line with a previous study of Dörr et al. [20], where a peak of *P. clarkii* with mature ovarian eggs was observed when the water temperature was about 20 °C and hydrological levels were around −0.8 m. However, in this study, with a hydrological level of −0.8 m but a lower temperature, the number of sexually mature females decreased. Indeed, for female crayfish pre-spawning phase (onset of egg maturation) takes place in June, and then the spawning phase (egg maturity) occurs in August-September, when the temperature was higher, suggesting that in Lake Trasimeno the reproductive cycle of *P. clarkii* females can be more affected by temperature rather than the hydrological levels.

In crayfish, the condition indexes based on weight are a valuable support to investigate the health state of a population [59,60] Tw/B and HIw were lower in males than in females, also in relation to the lower weight of the abdomen and hepatopancreas. This finding is also in agreement with the data reported by Dörr et al. [59] for wild specimens in Lake Trasimeno. However, in the present study, the weight difference between males and females reached 25%, whereas Dörr et al. [59] observed a difference of approximately 10%. This difference may be likely related to the smaller total size of males compared to females. In this regard, the values of Tw/B and HIw are comparable with those reported by Dörr et al. [4,59] and confirm a stable health condition of the species, suggesting that the changes of the hydrological levels did not affect its health status. HIw can be used as a sensitive morpho-physiological health marker, dependent on water nutrition, reproduction and pollution [59,60]. Dörr and Scalici [4] revealed an inverse correlation between HIw and GSI, dependent on the presence and weight of mature gonads in females. On the other hand, in the present investigation, there was no significant correlation between both condition indexes. In particular, GSI showed three peaks, corresponding to the period of higher incidence of mature gonads in females (August, September and October 2018).

**Figure 6:**
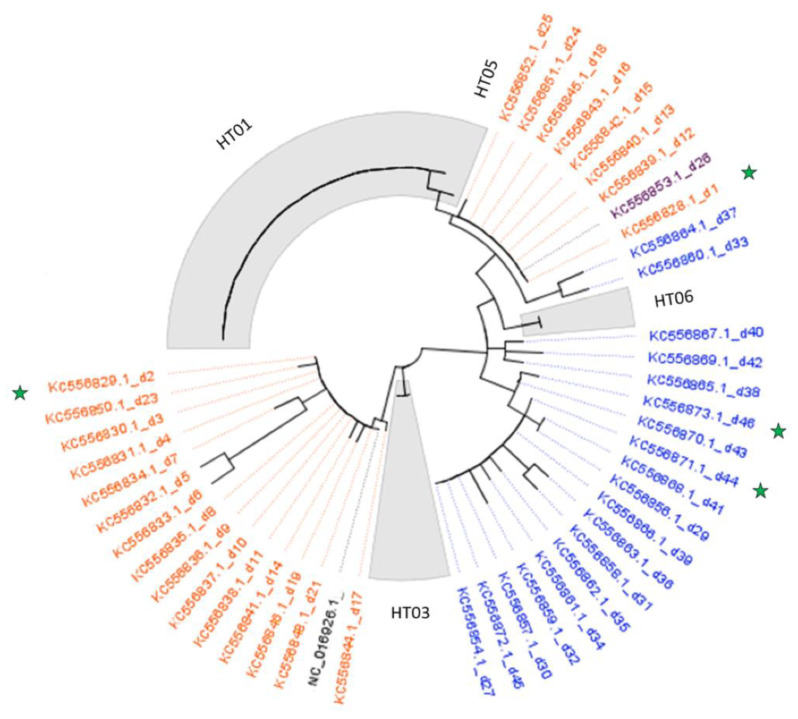
A phylogenetic tree that includes all samples from Lake Trasimeno (highlighted in grey) and sequences retrieved from GenBank (those considered ancestral haplotypes are marked with green stars). Haplotypes are shown in different colors according to the geographical regions from which the samples were collected: orange for China, purple for Japan and blue for USA.

Population genetic analyses can be helpful to assess origin, pathways of introduction and demographic changes associated with the dispersal of invasive species [40]. In order to ascertain the genetic affinities of our samples to other worldwide *P. clarkii* populations, the survey was extended to all available red swamp crayfish mitochondrial control region sequences. Unfortunately, to date, the only data concerning non-coding mtDNA are those published by Li et al. [40], who, by considering 291 individuals from 37 different sites of Asia and North America, identified a total of 46 haplotypes, including the four haplotypes inferred to be ancestral (Hap_d1, Hap_d2, Hap_d44, and Hap_d46). None of these four haplotypes was detected in our dataset, thus suggesting that the Lake Trasimeno population could be unique (Figure 6). Nevertheless, our four haplotypes were widely distributed in the precedent phylogeny, showing a clear separation of HT03, which falls in the exclusively Chinese clade, and the other haplotypes (HT01, HT05 and HT06), which belong to the other clade.

Indeed, the present study implemented the historical scenario based on mtDNA control region reported by Li et al. [40], by inferring *P. clarkii* population genetics and invasion routes in Europe. Previous investigations were undertaken in the European continent, Italy included, using the same population genetic approach, but focusing only on coding mtDNA markers, mainly 16S [61] and CO1 [28,31]. These previously published data are not consistent with the present study because it is based on the most polymorphic region of the mtDNA genome. Similarly, to the investigated control region haplotypes (HT01, HT05 and HT06, but not for HT03), all CO1 haplotypes found in European basins were closely related, differing by very few mutational steps. The authors argued that this low level of sequence divergence was expected, showing that the colonization of *P. clarkii* is recent [28]. It is evident that the Trasimeno’s population is distinct from those of other continents because the haplotypes were unique when compared to those already known [40], suggesting complex invasion dynamics followed by transfer and expansion. The marked prevalence of one haplotype (HT01) suggests a single introduction of *P. clarkii* into Lake Trasimeno, but the divergence of HT03 can strengthen the hypothesis of multiple secondary introductions as reported in the whole European continent [31]. Undoubtedly, further genetic investigations are needed by extending the dataset from Lake Trasimeno and other basins.

## 5. Conclusions

Morphometric and condition indexes indicate a good health state of both sexes of crayfish. The breeding period in Lake Trasimeno seems to be strongly related to water temperature and fluctuates with the hydrological level of the lake. In females, the pre-spawning phase is in June, and the spawning phase is from August to October. The frequency of sexually active males is generally high during all months of sampling and reaches its maximum value in the warmer summer months. Despite the imbalanced sex ratio showing a higher number of males than females, our results confirmed that *P. clarkii* has a stable population structure with decent condition indexes for both sexes and is skilled to deal with fluctuating water temperatures and levels and is favored by the resources and conditions typical of the Trasimeno ecosystem, which seems to be optimal for the maintenance of the species. As a consequence, almost 20 years after its first report, *P. clarkii* is still well acclimatized in the Trasimeno area. It is noteworthy that all mitochondrial haplotypes were unique when compared to those already known. In addition, the prevailing of a mitochondrial haplotype (HT01) suggests that a single predominant introduction event may have occurred in this area, likely followed by secondary events. Overall, the biology and genetic diversity investigation of *P. clarkii* provided relevant information which could be valuable for the management and control of this invasive species.

## Figures and Tables

**Table 1:** Seasonal chemical-physical parameters of Lake Trasimeno during the biomonitoring period (mean value ± standard deviation).

Variable	Summer	Autumn	Winter	Spring
water temperature (°C)	24.54 ± 4.34	10.52 ± 5.35	7.80 ± 2.94	21.17 ± 5.15
water transparency (m)	0.60 ± 0.10	0.52 ± 0.24	0.50 ± 0.17	0.83 ± 0.15
pH	8.54 ± 0.15	8.34 ± 0.06	8.42 ± 0.12	8.57 ± 0.19
dissolved oxygen (mg/L)	8.4 ± 1.02	10.42 ± 1.61	11.20 ± 1.02	8.13 ± 0.98
oxygen saturation (%)	97.12 ± 19.91	92.83 ± 3.58	94.00 ± 1.81	91.23 ± 3.83
conductivity (µS/cm)	1569.75 ± 116.80	1143.67 ± 192.55	1032.10 ± 89.06	1425.00 ± 167.82

**Table 2:** Monthly values of morphometric parameters of *P. clarkia* (mean value ± standard deviation).

	Total Length (mm)		Carapace Length (mm)		Total Weight (g)		Abdomen Weight (g)		Hepatopancreas Weight(g)	
Month	Females	Males	Females	Males	Females	Males	Females	Males	Females	Males
July 2018	107.02 ± 14.60	90.65 ± 12.14	52.76 ± 9.42	48.45 ± 5.71	31.50 ± 13.32	26.05 ± 9.60	8.56 ± 2.51	6.46 ± 1.85	1.76 ± 0.66	1.18 ± 0.49
August 2018	99.27 ± 11.39	88.34 ± 8.38	50.20 ± 7.04	43.80 ± 5.24	24.76 ± 10.44	19.37 ± 5.97	6.40 ± 2.10	4.80 ± 1.17	1.47 ± 0.55	0.95 ± 0.31
September 2018	94.88 ± 9.15	87.55 ± 8.78	47.38 ± 4.51	43.31 ± 4.56	20.83 ± 6.71	17.60 ± 5.52	5.38 ± 1.58	4.31 ± 1.22	1.43 ± 0.50	0.84 ± 0.25
October 2018	94.77 ± 11.50	92.88 ± 9.34	46.97 ± 5.46	46.25 ± 5.23	20.22 ± 7.75	21.50 ± 7.60	5.45 ± 2.00	4.92 ± 1.37	1.50 ± 0.76	1.20 ± 0.43
November 2018	90.66 ± 12.31	91.49 ± 10.61	44.59 ± 6.38	45.76 ± 5.82	17.81 ± 8.33	20.56 ± 9.26	4.83 ± 1.88	4.72 ± 1.56	1.37 ± 0.87	1.25 ± 0.60
December 2018	99.55 ± 13.22	94.99 ± 8.86	48.90 ± 6.70	47.50 ± 4.93	23.66 ± 10.39	23.63 ± 8.18	5.94 ± 2.24	5.13 ± 1.29	1.87 ± 1.01	1.60 ± 0.62
January 2019	93.26 ± 10.81	91.47 ± 10.40	45.43 ± 5.85	45.31 ± 5.66	18.71 ± 7.77	20.07 ± 9.05	5.01 ± 1.70	4.75 ± 1.63	1.45 ± 0.66	1.24 ± 0.50
February 2019	93.00 ± 10.25	92.29 ± 10.56	45.63 ± 5.47	45.92 ± 5.69	18.96 ± 7.98	20.75 ± 9.33	5.15 ± 1.67	4.82 ± 1.48	1.53 ± 0.87	1.31 ± 0.61
March 2019	103.70 ± 13.71	99.08 ± 9.04	50.24 ± 7.10	48.62 ± 5.02	25.85 ± 11.91	24.54 ± 7.99	6.70 ± 2.42	5.67 ± 1.56	1.97 ± 0.95	1.40 ± 0.48
April 2019	99.08 ± 12.15	97.98 ± 8.45	47.81 ± 6.49	47.92 ± 4.67	21.28 ± 9.35	22.69 ± 8.48	6.16 ± 2.17	5.57 ± 1.41	1.46 ± 0.76	1.34 ± 0.37
May 2019	103.53 ± 12.93	103.03 ± 9.59	50.00 ± 6.57	50.70 ± 5.36	24.01 ± 9.62	27.97 ± 10.33	7.04 ± 2.52	6.41 ± 1.62	1.73 ± 0.78	1.43 ± 0.47
June 2019	110.83 ± 10.40	106.92 ± 8.77	54.06 ± 5.31	53.02 ± 5.47	31.14 ± 10.40	33.13 ± 11.24	8.49 ± 2.36	7.24 ± 1.79	1.79 ± 0.63	1.47 ± 0.51
July 2019	111.69 ± 9.86	102.64 ± 9.89	54.33 ± 5.61	50.22 ± 5.52	30.53 ± 9.45	27.79 ± 9.47	8.84 ± 2.24	6.79 ± 1.72	1.94 ± 0.71	1.13 ± 0.41

**Table 3:** Control region mtDNA variation: haplotypes are shown with the respective mutations (within the range nps 4717–5434).

Sample ID	Mutations (Range: nps 4717–5434)	Haplotype Code
TR01	4752 4771 4865 4880 5033 5041 5056 5334 5335 5363 5370 5418	HT01
TR02	4752 4771 4865 4880 5033 5041 5056 5334 5335 5363 5370 5418	HT01
TR03	4752 4771 4865 4880 5033 5056 5334 5335 5363 5370 5418	HT05
TR04	4752 4771 4865 4880 5033 5041 5056 5334 5335 5363 5370 5418	HT01
TR05	4752 4771 4865 4880 5033 5041 5056 5334 5335 5363 5370 5418	HT01
TR06	4752 4771 4865 4880 5033 5041 5056 5334 5335 5363 5370 5418	HT01
TR07	4752 4771 4986 5033 5056 5334 5335 5352 5363 5370	HT06
TR08	4752 4771 4865 4880 5033 5041 5056 5334 5335 5363 5370 5418	HT01
TR09	4752 4771 4865 4880 5033 5041 5056 5334 5335 5363 5370 5418	HT01
TR10	4752 4771 4865 4880 5033 5041 5056 5334 5335 5363 5370 5418	HT01
TR11	4752 4771 4865 4880 5033 5041 5056 5334 5335 5363 5370 5418	HT01
TR12	4752 4771 4865 4880 5033 5041 5056 5334 5335 5363 5370 5418	HT01
TR13	4752 4771 4865 4880 5033 5041 5056 5334 5335 5363 5370 5418	HT01
TR14	4752 4771 4986 5033 5056 5334 5335 5352 5363 5370	HT06
TR15	4752 4771 4865 4880 5033 5041 5056 5334 5335 5363 5370 5418	HT01
TR16	4752 4771 4865 4880 5033 5041 5056 5334 5335 5363 5370 5418	HT01
TR17	5254 5334 5335 5363	HT03
TR18	4752 4771 4865 4880 5033 5041 5056 5334 5335 5363 5370 5418	HT01
TR19	5254 5334 5335 5363	HT03
TR20	4752 4771 4865 4880 5033 5041 5056 5334 5335 5363 5370 5418	HT01
TR21	4752 4771 4865 4880 5033 5041 5056 5334 5335 5363 5370 5418	HT01
TR22	5254 5334 5335 5363	HT03
TR23	4752 4771 4865 4880 5033 5041 5056 5334 5335 5363 5370 5418	HT01
TR24	4752 4771 4865 4880 5033 5041 5056 5334 5335 5363 5370 5418	HT01
TR25	4752 4771 4865 4880 5033 5041 5056 5334 5335 5363 5370 5418	HT01
TR26	4752 4771 4865 4880 5033 5041 5056 5334 5335 5363 5370 5418	HT01
TR27	4752 4771 4865 4880 5033 5041 5056 5334 5335 5363 5370 5418	HT01
TR28	4752 4771 4865 4880 5033 5041 5056 5334 5335 5363 5370 5418	HT01
TR29	5254 5334 5335 5363	HT03

## Data Availability

Additional datasets generated and analyzed in this study are available upon request to the corresponding author.

## References

[1] Hobbs H.H., Holdich D.M., Lowery R.S. (1988). Crayfish Distribution, Adaptive Radiation and Evolution. Freshwater Crayfish: Biology, Management and Exploitation.

[2] Gherardi F. (2006). Crayfish Invading Europe: The Case Study of *Procambarus clarkii*. Mar. Freshw. Behav. Physiol..

[3] Claire W.H., Wroiten J.W. (1978). First Record of the Crayfish, *Procambarus clarkii*, from Idaho, USA (Decapoda, Cambaridae). Crustaceana.

[4] Dörr A.J.M., Scalici M. (2013). Revisiting Reproduction and Population Structure and Dynamics of *Procambarus clarkii* Eight Years after Its Introduction into Lake Trasimeno (Central Italy). Knowl. Manag. Aquat. Ecosyst..

[5] Xiao Q., Zhang M.-T., Wu Y., Ding H., Lei J.-C., Zhu S.-L., Zhang Z.-H., Chen L. (2020). Prediction of Potential Distribution of the Invasive Species *Procambarus clarkii* in China Based on Ecological Niche Models. J. Appl. Ecol..

[6] Piersanti S., Pallottini M., Salerno G., Goretti E., Elia A.C., Dörr A.J.M., Rebora M. (2018). Resistance to Dehydration and Positive Hygrotaxis in the Invasive Red Swamp Crayfish *Procambarus clarkii*. Knowl. Manag. Aquat. Ecosyst..

[7] Zanotto F.P., Wheatly M.G. (1993). The Effect of Ambient PH on Electrolyte Regulation during the Postmoult Period in Freshwater Crayfish *Procambarus clarkii*. J. Exp. Biol..

[8] Dörr A.J.M., Scalici M., Caldaroni B., Magara G., Scoparo M., Goretti E., Elia A.C. (2020). Salinity Tolerance of the Invasive Red Swamp Crayfish *Procambarus clarkii* (Girard, 1852). Hydrobiologia.

[9] Scalici M., Chiesa S., Scuderi S., Celauro D., Gibertini G. (2010). Population Structure and Dynamics of *Procambarus clarkii* (Girard, 1852) in a Mediterranean Brackish Wetland (Central Italy). Biol. Invasions.

[10] Elia A.C., Dörr A.J.M., Mastrangelo C., Prearo M., Abete M.C. (2006). Glutathione and Antioxidant Enzymes in the Hepatopancreas of Crayfish *Procambarus clarkii* (Girard, 1852) of Lake Trasimeno (Italy). Bull. Fr. Pêche Piscic..

[11] Kouba A., Buřič M., Kozák P. (2010). Bioaccumulation and Effects of Heavy Metals in Crayfish: A Review. Water Air Soil Pollut..

[12] Trombini C., Kazacova J., Montilla-López A., Fernández-Cisnal R., Hampel M., Fernández-Torres R., Bello-López M.Á., Abril N., Blasco J. (2021). Assessment of Pharmaceutical Mixture (Ibuprofen, Ciprofloxacin and Flumequine) Effects to the Crayfish *Procambarus clarkii*: A Multilevel Analysis (Biochemical, Transcriptional and Proteomic Approaches). Environ. Res..

[13] Alcorlo P., Baltanás A. (2013). The Trophic Ecology of the Red Swamp Crayfish (*Procambarus clarkii*) in Mediterranean Aquatic Ecosystems: A Stable Isotope Study. Limnetica.

[14] Mancinelli G., Papadia P., Ludovisi A., Migoni D., Bardelli R., Fanizzi F.P., Vizzini S. (2018). Beyond the Mean: A Comparison of Trace-and Macroelement Correlation Profiles of Two Lacustrine Populations of the Crayfish *Procambarus clarkii*. Sci. Total Environ..

[15] Dörr A.J., Elia A.C., Rodolfi M., Garzoli L., Picco A.M., D’Amen M., Scalici M. (2012). A Model of Co-Occurrence: Segregation and Aggregation Patterns in the Mycoflora of the Crayfish *Procambarus clarkii* in Lake Trasimeno (Central Italy). J. Limnol..

[16] Dörr A.J., Rodolfi M., Scalici M., Elia A.C., Garzoli L., Picco A.M. (2011). Phoma Glomerata, a Potential New Threat to Italian Inland Waters. J. Nat. Conserv..

[17] Souty-Grosset C., Anastacio P.M., Aquiloni L., Banha F., Choquer J., Chucholl C., Tricarico E. (2016). The Red Swamp Crayfish *Procambarus clarkii* in Europe: Impacts on Aquatic Ecosystems and Human Well-Being. Limnologica.

[18] Veroli M., Martinoli M., Caprioli R., Angelici C., Pulcini D., Capoccioni F. (2021). Population Structure and Dynamics of the Invasive *Procambarus clarkii* (Girard, 1852) in a Tiber River Ramsar Site, Central Italy. Int. J. Aquat. Biol..

[19] Delmastro G.B. (1992). Sull’acclimatazione Del Gambero Della Louisiana *Procambarus clarkii* (Girard, 1852) Nelle Acque Dolci Italiane (Crustacea: Decapoda: Cambaridae). Pianura.

[20] Dörr A.J.M., La Porta G., Pedicillo G., Lorenzoni M. (2006). Biology of *Procambarus clarkii* (Girard, 1852) in Lake Trasimeno. Bull. Fr. Pêche Piscic..

[21] Frondini F., Dragoni W., Morgantini N., Donnini M., Cardellini C., Caliro S., Melillo M., Chiodini G. (2019). An Endorheic Lake in a Changing Climate: Geochemical Investigations at Lake Trasimeno (Italy). Water.

[22] Ludovisi A., Gaino E. (2010). Meteorological and Water Quality Changes in Lake Trasimeno (Umbria, Italy) during the Last Fifty Years. J. Limnol..

[23] Coops H., Beklioglu M., Crisman T.L. (2003). The Role of Water-Level Fluctuations in Shallow Lake Ecosystems–Workshop Conclusions. Hydrobiologia.

[24] Souty-Grosset C., Holdich D., Noel P., Reynolds J.D., Haffner P. (2006). Atlas of Crayfish in Europe.

[25] Alcorlo P., Geiger W., Otero M. (2008). Reproductive Biology and Life Cycle of the Invasive Crayfish *Procambarus clarkii* (Crustacea: Decapoda) in Diverse Aquatic Habitats of South-Western Spain: Implications for Population Control. Fundam. Appl. Limnol..

[26] Reynolds J.D. (2002). Growth and Reproduction. Biology of Freshwater Crayfish.

[27] Acevedo-Limón L., Oficialdegui F.J., Sánchez M.I., Clavero M. (2020). Historical, Human, and Environmental Drivers of Genetic Diversity in the Red Swamp Crayfish (*Procambarus clarkii*) Invading the Iberian Peninsula. Freshw. Biol..

[28] Barbaresi S., Gherardi F., Mengoni A., Souty-Grosset C. (2007). Genetics and invasion biology in fresh waters: A pilot study of *Procambarus clarkii* in Europe. Biological Invaders in Inland Waters: Profiles, Distribution, and Threats.

[29] Loureiro T.G., Anastácio P.M.S.G., Araujo P.B., Souty-Grosset C., Almerão M.P. (2015). Red Swamp Crayfish: Biology, Ecology and Invasion-an Overview. Nauplius.

[30] Oficialdegui F.J., Sánchez M.I., Clavero M. (2020). One Century Away from Home: How the Red Swamp Crayfish Took over the World. Rev. Fish Biol. Fish..

[31] Oficialdegui F.J., Clavero M., Sánchez M.I., Green A.J., Boyero L., Michot T.C., Klose K., Kawai T., Lejeusne C. (2019). Unravelling the Global Invasion Routes of a Worldwide Invader, the Red Swamp Crayfish (*Procambarus clarkii*). Freshw. Biol..

[32] Li Y., Wang W., Liu X., Luo W., Zhang J., Gul Y. (2011). DNA Extraction from Crayfish Exoskeleton. Indian J. Exp. Biol..

[33] Yi S., Li Y., Shi L., Zhang L., Li Q., Chen J. (2018). Characterization of Population Genetic Structure of Red Swamp Crayfish, *Procambarus clarkii*, in China. Sci. Rep..

[34] Zhong Y., Tang Z., Huang L., Wang D., Lu Z. (2020). Genetic Diversity of *Procambarus clarkii* Populations Based on Mitochondrial DNA and Microsatellite Markers in Different Areas of Guangxi, China. Mitochondrial DNA Part A.

[35] Quan A.S., Pease K.M., Breinholt J.W., Wayne R.K. (2014). Origins of the Invasive Red Swamp Crayfish (*Procambarus clarkii*) in the Santa Monica Mountains. Aquat. Invasions.

[36] R Core Team (2021). R: A Language and Environment for Statistical Computing.

[37] Wickham H., François R., Henry L., Müller K. (2021). Dplyr: A Grammar of Data Manipulation. https://CRAN.R-project.org/package=dplyr.

[38] Wickham H. (2021). Tidyr: Tidy Messy Data. https://CRAN.R-project.org/package=tidyr.

[39] Wickham H. (2016). Ggplot2: Elegant Graphics for Data Analysis.

[40] Li Y., Guo X., Chen L., Bai X., Wei X., Zhou X., Huang S., Wang W. (2015). Inferring Invasion History of Red Swamp Crayfish (*Procambarus clarkii*) in China from Mitochondrial Control Region and Nuclear Intron Sequences. Int. J. Mol. Sci..

[41] Kim S., Park M.-H., Jung J.-H., Ahn D.-H., Sultana T., Kim S., Park J.-K., Choi H.-G., Min G.-S. (2012). The Mitochondrial Genomes of Cambaroides Similis and *Procambarus clarkii*(Decapoda: Astacidea: Cambaridae): The Phylogenetic Implications for Reptantia. Zool. Scr..

[42] Librado P., Rozas J. (2009). DnaSP v5: A Software for Comprehensive Analysis of DNA Polymorphism Data. Bioinformatics.

[43] Huner J.V. (2002). Procambarus. Biology of Freshwater Crayfish.

[44] Oluoch A.O. (1990). Breeding Biology of the Louisiana Red Swamp Crayfish *Procambarus clarkii* Girard in Lake Naivasha, Kenya. Hydrobiologia.

[45] Vogt G. (2013). Abbreviation of Larval Development and Extension of Brood Care as Key Features of the Evolution of Freshwater Decapoda. Biol. Rev..

[46] Elia A.C., Dörr A.J.M., Abete M.C., Prearo M. (2010). Seasonal Variability of Detoxificant Response and Heavy Metal Accumulation in Tissues of Both Sexes in *Tinca tinca* (L.) from Lake Trasimeno. Rev. Fish Biol. Fish..

[47] Donato R., Rollandin M., Favaro L., Ferrarese A., Pessani D., Ghia D. (2018). Habitat Use and Population Structure of the Invasive Red Swamp Crayfish *Procambarus clarkii* (Girard, 1852) in a Protected Area in Northern Italy. Knowl. Manag. Aquat. Ecosyst..

[48] Ligas A. (2008). Population Dynamics of *Procambarus clarkii* (Girard, 1852) (Decapoda, Astacidea, Cambaridae) from Southern Tuscany (Italy). Crustaceana.

[49] Maccarrone V., Filiciotto F., Buffa G., Di Stefano V., Quinci E.M., de Vincenzi G., Mazzola S., Buscaino G. (2016). An Invasive Species in a Protected Area of Southern Italy: The Structure, Dynamics and Spatial Distribution of the Crayfish *Procambarus clarkii*. Turk. J. Fish. Aquat. Sci..

[50] Gherardi F., Tricarico E., Ilhéu M. (2002). Movement Patterns of an Invasive Crayfish, *Procambarus clarkii*, in a Temporary Stream of Southern Portugal. Ethol. Ecol. Evol..

[51] Gherardi F., Barbaresi S., Salvi G. (2000). Spatial and Temporal Patterns in the Movement of *Procambarus clarkii*, an Invasive Crayfish. Aquat. Sci..

[52] Gherardi F., Barbaresi S. (2000). Invasive Crayfish: Activity Patterns of *Procambarus clarkii* in the Rice Fields of the Lower Guadalquivir (Spain). Arch. Für Hydrobiol..

[53] Bravo M.A., Duarte C.M., Montes C. (1994). Environmental Factors Controlling the Life History of *Procambarus clarkii* (Decapoda, Cambaridae) in a Temporary Marsh of the Doñana National Park (SW Spain). Int. Ver. Für Theor. Angew. Limnol. Verh..

[54] Huner J.V., Holdich D.M., Lowery R.S. (1988). Procambarus in North America and Elsewhere. Freshwater Crayfish: Biology, Management and Exploitation.

[55] Penn G.H. (1943). A Story of the Life History of the Louisiana Crayfish *Procambarus clarkii* (Girard). Ecology.

[56] Guerra J.L., Niño A.E. (1995). Ecology of Red Swamp Crayfish (*Procambarus clarkii*, Girard) in the Central Meseta of Spain. Freshw. Crayfish.

[57] Dalosto M.M., Palaoro A.V., Souty-Grosset C., de Siqueira Bueno S.L., Loureiro T.G., Almerão M.P., de Araujo P.B., Santos S. (2015). One Step Ahead of the Enemy: Investigating Aggressive Interactions between Invasive and Native Crayfish before the Contact in Nature. Biol. Invasions.

[58] Hanshew B.A., Garcia T.S. (2012). Invasion of the Shelter Snatchers: Behavioural Plasticity in Invasive Red Swamp Crayfish, *Procambarus clarkii*. Freshw. Biol..

[59] Dörr A.J.M., Abete M.C., Prearo M., Pacini N., La Porta G., Natali M., Elia A.C. (2013). Effects of Selenium Supplemented Diets on Growth and Condition Indexes in Juvenile Red Swamp Crayfish, *Procambarus clarkii*. Environ. Toxicol. Pharmacol..

[60] Evans L.H., Jussila J. (1997). Freshwater Crayfish Growth under Culture Conditions: Proposition for a Standard Reporting Approach. J. World Aquac. Soc..

[61] Barbaresi S., Fani R., Gherardi F., Mengoni A., Souty-Grosset C. (2003). Genetic Variability in European Populations of an Invasive American Crayfish: Preliminary Results. Biol. Invasions.

